# Possible role of chondroitin sulphate and glucosamine for primary prevention of colorectal cancer. Results from the MCC-Spain study

**DOI:** 10.1038/s41598-018-20349-6

**Published:** 2018-02-01

**Authors:** Gemma Ibáñez-Sanz, Anna Díez-Villanueva, Laura Vilorio-Marqués, Esther Gracia, Nuria Aragonés, Rocío Olmedo-Requena, Javier Llorca, Juana Vidán, Pilar Amiano, Pilar Nos, Guillermo Fernández-Tardón, Ricardo Rada, María Dolores Chirlaque, Elisabet Guinó, Verónica Dávila-Batista, Gemma Castaño-Vinyals, Beatriz Pérez-Gómez, Benito Mirón-Pozo, Trinidad Dierssen-Sotos, Jaione Etxeberria, Amaia Molinuevo, Begoña Álvarez-Cuenllas, Manolis Kogevinas, Marina Pollán, Victor Moreno

**Affiliations:** 1 Unit of Biomarkers and Susceptibility, Cancer Prevention and Control Programme, Catalan Institute of Oncology-IDIBELL, L’Hospitalet de Llobregat, Spain; 2 Gastroenterology Department, Bellvitge University Hospital-IDIBELL, L’Hospitalet de Llobregat, Spain; 3 Grupo de Investigación en Interacciones Gen-Ambiente y Salud, Universidad de León, León, Spain; 4 CIBER Epidemiología y Salud Pública (CIBERESP), Madrid, Spain; 5 ISGlobal Centre for Research in Environmental Epidemiology (CREAL), Barcelona, Spain; 6 Universitat Pompeu Fabra (UPF), Barcelona, Spain; 7 Environmental and Cancer Epidemiology Department, National Centre of Epidemiology - Instituto de Salud Carlos III, Madrid, Spain; 8 Oncology and Hematology Area, IIS Puerta De Hierro, Cancer Epidemiology Research Group, Madrid, Spain; 9 Instituto de Investigación Biosanitaria de Granada (ibs.GRANADA), Servicio Andaluz de Salud/Universidad de Granada, Granada, Spain; 10 Departamento de Medicina Preventiva y Salud Pública, Universidad de Granada, Granada, Spain; 11 Universidad de Cantabria - IDIVAL, Santander, Spain; 12 Navarra Public Health Institute, Pamplona, Spain; 13 IdiSNA, Navarra Institute for Health Research, Pamplona, Spain; 14 Public Health Division of Gipuzkoa, Biodonostia Research Institute, San Sebastian, Spain; 15 La Fe University and Politechnic Hospital, Health Research Institute La Fe, Valencia, Spain; 16 CIBER Enfermedades hepáticas y digestivas (CIBEREHD), Madrid, Spain; 17 University Institute of Oncology of Asturias (IUOPA), Universidad de Oviedo, Oviedo, Spain; 18 Juan Ramon Jiménez University Hospital, University of Huelva, Huelva, Spain; 19 Centre for Research in Health and Environment (CYSMA), University of Huelva, Huelva, Spain; 20 Department of Epidemiology, Murcia Regional Health Council, IMIB-Arrixaca and Department of Health and Social Sciences, Universidad de Murcia, Murcia, Spain; 21 IMIM (Hospital del Mar Medical Research Institute), Barcelona, Spain; 22 Unidad de Gestión de Cirugía. Complejo Hospitalario Universitario de Granada, Granada, Spain; 23 Department of Statistics and O. R., Public University of Navarre, Navarre, Spain; 24 Gastroenterology Department, Complejo Asistencial Universitario de León, León, Spain; 25 School of Public Health, Athens, Greece; 26 Department of Clinical Sciences, Faculty of Medicine, University of Barcelona, Barcelona, Spain

## Abstract

A safe and effective colorectal cancer (CRC) chemoprevention agent remains to be discovered. We aim to evaluate the association between the use of glucosamine and/or chondroitin sulphate and risk of colorectal cancer (CRC) in the MCC-Spain study, a case-control study performed in Spain that included 2140 cases of CRC and 3950 population controls. Subjects were interviewed on sociodemographic factors, lifestyle, family and medical history and regular drug use. Adjusted odds ratios and their 95% confidence intervals were estimated. The reported frequency of chondroitin and/or glucosamine use was 2.03% in controls and 0.89% in cases. Users had a reduced risk of CRC (OR: 0.47; 95% CI: 0.28–0.79), but it was no longer significant when adjusted for NSAID (nonsteroidal anti-inflammatory drugs) use (OR: 0.82; 95% CI: 0.47–1.40). A meta-analysis with previous studies suggested a protective effect, overall and stratified by NSAID use (OR: 0.77; 95% CI: 0.62–0.97). We have not found strong evidence of an independent preventive effect of CG on CRC in our population because the observed effects of our study could be attributed to NSAIDs concurrent use. These results merit further research due to the safety profile of these drugs.

## Introduction

The high incidence of colorectal cancer (CRC), the known colorectal adenoma-to carcinoma sequence and the poor survival rate of advanced CRC has prompted the emphasis on its prevention. Faecal occult blood test has demonstrated a reduction of CRC mortality^1^. This strategy is based on early detection, and requires repeated testing to increase sensitivity. Although lifestyle risk factors have been described in CRC aetiology, randomized trials have failed to show a reduction of adenomas recurrence with special diets^2^. Moreover, a safe and effective CRC chemoprevention agent has not been found to date in order to reduce the incidence of polyps and/or CRC. Acetylsalicylic acid (ASA) is the agent with more evidence and, indeed, the United States Preventive Services Task Force^3^ has recently stated that there is adequate evidence^4^ that aspirin can be used to reduce risk for CRC in adults ages 50 to 69 years who are at increased risk for cardiovascular diseases. However, a general use of this drug in younger people or without cardiovascular disease is not recommended because of its adverse events such as gastrointestinal and intra-cerebral haemorrhage^5^. Other drugs and supplements have also been studied as candidate chemoprevention agents for CRC such as other non-steroidal anti-inflammatory drugs (NSAIDs)^6–8^, folic acid^9,10^, calcium^10,11^, and diverse vitamins^10,12^. None of them have shown enough evidence to be implemented as chemoprevention agents for general population.

Recent evidence suggests that glucosamine and chondroitin sulphate supplements could reduce CRC risk^13–15^. These drugs are widely used in osteoarthritis due to their immunomodulatory effect, which reduces the nuclear factor κβ (NF-kβ) translocation. NF-kβ has an established role in the coordination of innate and adaptive immune responses and cell-cycle regulation and it has a role in tumorigenesis^16^. In the VITamins And Lifestyle (VITAL)^13,14^ study and in two prospective cohorts, Kantor *et al*.^15^ reported that the use of these drugs had a protective effect of CRC risk. Moreover, the good tolerability of these drugs has been proven in trials that aimed to study the efficacy and safety of chondroitin sulphate plus glucosamine in osteoarthritis^17–19^. For this reason, we wanted to explore the association between glucosamine and chondroitin sulphate and CRC in the MCC-Spain case-control study.

## Methods

### Study population

A Multi Case-control (MCC-Spain) study was performed between 2008 and 2013, in which 10183 total subjects aged 20–85 years were enrolled in 12 Spanish provinces. A detailed description has been previously published^20^. The recruitment included incident cases of CRC (C18, C19, C20, D01.0, D01.1, D01.2) which were identified through an active search in the participating hospitals. Both cases and controls were free of personal CRC history. Controls, selected from the general population, were frequency-matched to cases, by age, sex, and region. In this study, we included 2140 cases of CRC and 3950 controls.

All procedures were in accordance with the ethical standards of the institutional and/or national research committee, and with the 1964 Helsinki Declaration and its later amendments or comparable ethical standards. The protocol of MCC-Spain was approved by each of the ethics committees of the participating institutions. The specific study reported here was approved by the Bellvitge Hospital Ethics Committee with reference PR149/08. Written informed consent was obtained from all individual participants included in the study.

### Data collection

A structured computerised epidemiological questionnaire was administered by trained personnel in a face-to-face interview. This questionnaire included information of sociodemographic factors, personal and family medical history, anthropometric data, lifestyle and medication. Also, subjects filled in a semi-quantitative Food Frequency Questionnaire (FFQ).

Complete history of regular drug use was recorded, obtained by personal interview, but only chondroitin sulphate (ATC code: M01AX25), glucosamine (ATC code: M01AX05) and NSAIDs including non-ASA NSAIDs (ATC code: M01A) and ASA (ATC code: B01AC06, N02BA01, NA02BA51) were considered for this study. For each drug, the brand name, dose and duration of exposure were recorded. Unless specified, we will refer to NSAIDs as the combination of ASA and other NSAIDs. Regular use NSAIDs was defined as consuming ≥1 times/day for at least one year. However, the low frequency of use of glucosamine or chondroitin sulphate only allowed dividing patients into users (either “regular” or “sporadic”) and nonusers. Given that chondroitin sulphate and glucosamine are frequently consumed combined and there were few individuals using these drugs, their combined use was analysed.

### Statistical analysis

A study design adjustment score (SDAS) was built to reduce bias related to differences in case and control selection frequencies. This SDAS was derived as the prediction of a logistic regression model on case-control status that included age, sex, recruiting centre and level of education and it also included the interactions between age and sex and centre and sex. All analyses were adjusted by the SDAS, and multivariable-adjusted analyses also included non-ASA NSAIDs and ASA use, family history of CRC, tobacco use, alcohol consumption, BMI (estimated at age 45 years), physical activity, red meat intake and vegetables intake. Stratified analyses were also performed to assess the association of chondroitin/glucosamine with CRC according to NSAIDs use and BMI, since previous studies suggested a possible interaction with these factors. Logistic regression models were used to test for adjusted effects and interactions. Results are reported as odds ratios (OR) and 95% confidence intervals (CI). All reported p-values are two-tailed. A fixed-effects meta-analysis was performed, combining the estimates from this study with previous published results^14,15^. Statistical analysis was carried out using R statistical software (R Foundation for Statistical Computing, Vienna, Austria).

## Results

Overall, 99 participants (1.63%) reported use of chondroitin sulphate (n = 60) and/or glucosamine (n = 45). Table 1 shows the characteristics of chondroitin sulphate and glucosamine users versus non-users among controls. The use of these drugs was only associated with no-ASA NSAIDs consumption (p < 0.001). In contrast, its use was independent of sex, age, tobacco use, alcohol consumption, BMI, physical activity, intake of vegetables or red meat, and ASA prescription. Regarding other drugs used to treat osteoarthritis, we observed that concurrent use of chondroitin sulphate and glucosamine with non-ASA NSAIDs was around 84.9% but with ASA was only 14.1%. In fact, 98% participants consumed non-ASA NSAIDs as an analgesic drug (30.7% for joint pain and 67.8% for pain in other locations); 91.7% and 87.5% consumed chondroitin sulphate and glucosamine for a joint disease, respectively; and 62.9% subjects were prescribed ASA for primary or secondary prevention of cardiovascular events.

**Table 1:** Characteristics of the chondroitin sulphate and glucosamine users in controls.

Characteristic	Nonusers^a^		Users		P-value^b^
	n	(%)	n	(%)	
**Age (years)**					
26–65	2066	(53.4)	39	(48.8)	
65–85	1804	(46.6)	41	(51.3)	0.41
**Sex**					
Female	1886	(48.7)	46	(57.5)	
Male	1984	(51.3)	34	(42.5)	0.12
**Smoking**					
Non-smoker	1721	(44.5)	42	(52.5)	
Former/Current smoker	2149	(55.5)	38	(47.5)	0.15
**Alcohol**					
Low consumption	3315	(85.7)	66	(82.5)	
High consumption	555	(14.3)	14	(17.5)	0.43
BMI at 45-year age					
<25kg/m^2^	2225	(57.5)	54	(67.5)	
≥25kg/m^2^	1645	(42.5)	26	(32.5)	0.80
**Physical activity in leisure time**					
No	1623	(41.9)	26	(32.5)	
Yes	2247	(58.1)	54	(67.5)	0.09
Vegetables					
≤200g/day	2564	(66.3)	54	(67.5)	
>200g/day	1306	(33.8)	26	(32.5)	0.81
Red meat					
≤65g/day	2269	(58.6)	52	(65.0)	
>65g/day	1601	(41.4)	28	(35.0)	0.25
**ASA**					
Nonuser/sporadically use	3386	(87.5)	66	(57.5)	
Regular use in the last year	484	(12.5)	14	(17.5)	0.19
**Non-ASA NSAIDs**					
Nonuser/sporadically use	3200	(82.7)	11	(13.8)	
Regular use in the last year	670	(17.3)	69	(86.3)	<0.0001

^a^User includes sporadically use and regular use.

^b^P-values derived from a chi-square test.

ASA: acetylsalicylic acid; BMI: body mass index; CRC: colorectal cancer; NSAID: Nonsteroidal anti-inflammatory drugs.

In the crude analysis (only adjusted for the SDAS), chondroitin sulphate and/or glucosamine (CG) use was associated with a 53% reduced risk of CRC (OR: 0.47; 95% CI: 0.28–0.79). Both the use of chondroitin sulphate alone (OR: 0.42; 95% CI: 0.21–0.84) and glucosamine alone (OR: 0.47, 95% CI: 0.22–1.01) were protective for CRC.

In the multivariate-adjusted analysis (Table 2), the use of CG was not significantly associated with CRC (adjusted OR: 0.82; 95% CI: 0.47–1.40), probably due to the small number of exposed that limited power, and the concurrent use of NSAIDs. Regular use of ASA and non-ASA NSAIDs significantly reduced CRC risk by 25–43% in the MCC-Spain study (adjusted OR 0.75; 95% CI: 0.63–0.90, and adjusted OR: 0.54; 95% CI: 0.46–0.65, respectively). Table 3 shows how the protective effect was no longer significant when adjusted for NSAIDs use.

**Table 2:** Multivariate-adjusted risk factors associated with CRC.

Characteristic	Controls		Cases		Adjusted	95% CI	P-Value
	n	%	n	%	OR^a^		
**Family history of CRC**							
No	3483	(88.2)	1663	(77.7)	1.00		
Yes	467	(11.8)	477	(22.3)	2.43	2.09–2.83	<0.0001
**Smoking**							
Non-smoker	1763	(44.6)	893	(41.7)	1.00		
Former/Current smoker	2187	(55.8)	1247	(58.3)	1.05	0.93–1.84	0.43
**Alcohol**							
Low consumption	3381	(85.6)	1685	(78.7)	1.00		
High consumption	569	(14.4)	455	(21.3)	1.35	1.16–1.57	<0.0001
**BMI at 45-year age**							
<25kg/m2	2279	(57.7)	982	(45.9)	1.00		
≥25kg/m2	1671	(42.3)	1158	(54.1)	1.16	1.03–1.30	0.01
**Physical activity in leisure time**							
No	1649	(41.8)	1101	(51.5)	1.00		
Yes	2301	(58.3)	1039	(48.6)	0.70	0.63–0.78	<0.0001
**Vegetables**							
≤200g/day	2618	(66.3)	1526	(71.3)	1.00		
>200g/day	1332	(33.7)	614	(28.7)	0.75	0.66–0.85	<0.0001
**Red meat**							
≤65g/day	2321	(58.8)	1070	(50.0)	1.00		
>65g/day	1629	(41.2)	1070	(50.0)	1.22	1.09–1.38	0.0008
**ASA**							
Non-user/sporadically use	3452	(87.4)	1894	(88.5)	1.00		
Regular use in the last year	498	(12.6v	246	(11.5)	0.75	0.63–0.90	0.0013
**Non-ASA NSAIDs**							
Non-user/sporadically use	3181	(80.5)	1912	(89.4)	1.00		
Regular use in the last year	769	(19.5v	228	(10.7)	0.54	0.46–0.65	<0.0001
**Chondroitin and/or glucosamine**							
Non-user	3870	(98.0)	2121	(99.1)	1.00		
Userb	80	(2.0v	19	(0.9)	0.82	0.47–1.40	0.37

^a^Adjusted by the study design adjustment and the variables shown in this table.

ASA: acetylsalicylic acid; BMI: Body mass index; CRC: colorectal cancer; NSAID: Nonsteroidal anti-inflammatory drugs.

**Table 3:** Chondroitin sulphate and glucosamine association to risk of CRC.

	OR	95% CI	P-value
Crude effect^a^	0.47	0.28–0.79	0.0023
Adjusted for ASA use	0.47	0.28–0.79	0.0045
Adjusted for non-ASA NSAIDs use	0.72	0.43–1.23	0.23
Adjusted for NSAIDs use	0.62	0.37–1.05	0.077
Adjusted for multivariate^b^ without NSAIDs use	0.52	0.31–0.88	0.017
Adjusted for multivariate^b^	0.82	0.47–1.40	0.37

^a^Adjusted by the study design variables (age, gender, region and education).

^b^Adjusted by the study design variables plus alcohol consumption, BMI, physical activity, vegetables and red meat intake, family history and NSAIDs use (see Table 3).

ASA: acetylsalicylic acid; NSAID: Nonsteroidal anti-inflammatory drugs (includes ASA except when indicated).

The combined analysis (Table 4) showed that the protective effect of CG on CRC was only among subjects that were NSAIDs users. An increased protective effect with concurrent use of CG and NSAIDs was found, suggesting a possible additive action.

**Table 4:** Analysis of chondroitin and/or glucosamine protective effect of CRC according to NSAID use

	Control			Case			
Interaction analysis between chondroitin and/or glucosamine use and NSAIDs							
	n	%	n	%	Adjusted OR^a^	95% CI	
CG nonuser^b^ - NSAIDs nonuser^c^	2776	70.28	1697	79.30	1.00		
CG user - NSAIDs nonuser	10	0.250	4	0.19	1.04	0.31–3.55	
CG nonuser - NSAIDs user	1094	27.70	414	19.81	0.62	0.53–0.71	
CG user - NSAIDs user	70	1.77	15	0.70	0.40	0.22–0.72	
**Stratified analysis of chondroitin and/or glucosamine protective effect of CRC according to NSAID use**							
	**n**	**%**	**n**	**%**	**Adjusted OR** ^**a**^	**95% CI**	**p-interaction**
NSAIDs nonuser							0.50
CG nonuser	2803	99.64	1715	99.77	1		
CG user	10	0.36	4	0.23	1.04	0.30–3.54	
**NSAIDs user** ^**c**^							
CG nonuser	1067	93.84	406	96.44			
CG user	70	6.16	15	3.56	0.66	0.37–1.21	

^a^Adjusted by the study design variables (age, gender, region and education) plus alcohol consumption, BMI, physical activity, vegetables and red meat intake, family history and NSAIDs.

^b^User includes sporadically use and regular use.

^c^User includes only regular use of NSAIDs.

CG: chondroitin and/or glucosamine. NSAID: Nonsteroidal anti-inflammatory drugs (including ASA).

**Figure 1:**
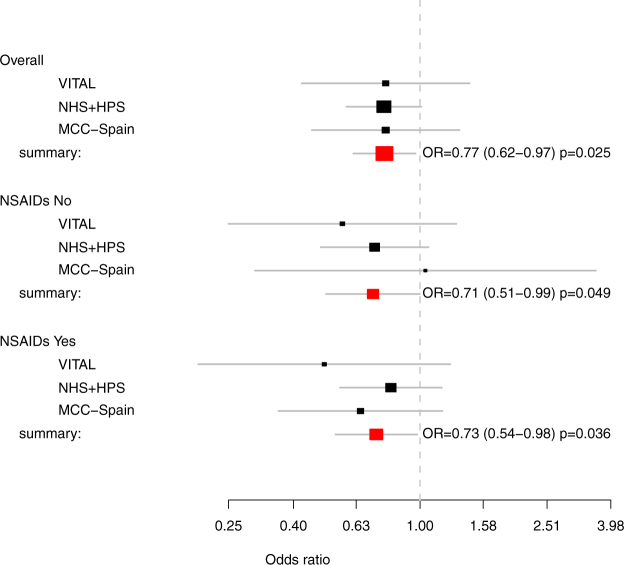
Meta-analysis of studies of chondroitin sulphate and glucosamine and the risk of CRC. Estimated heterogeneity variance = 0.01, P = 0.99.

The fixed-effects meta-analysis of the multivariate-adjusted estimates, both overall (OR 0.77; 95%CI: 0.62–0.97; p = 0.025) and stratified by NSAID use (OR for NSAID users 0.73; 95%CI: 0.54–0.98; p = 0.036. OR for non-NSAID users 0.71; 95%CI: 0.51–0.99; p = 0.049) confirmed a significant protective effect of CG in CRC (Fig. 1). No evidence of heterogeneity was observed (estimated heterogeneity variance = 0.01, p = 0.99; test for funnel plot asymmetry: z = 0.052, p = 0.96).

## Discussion

The results of this case-control study did not show clear evidence of a preventive effect of CG on CRC because, though in the univariate analysis CG had a significant association, this effect was no longer significant when adjusted for NSAID use. The number of subjects exposed to CG was low, and this reduced the power to detect a protective effect. The analysis stratified by NSAIDs use indicated that the effect of CG was additive to the concurrent use of these drugs (Table 4).

The OR for CG was no longer significant when adjusted for NSAIDs use but the magnitude of the adjusted effect was similar to that reported recently by Kantor *et al*.^15^ in two prospective cohorts in North America (RR: 0.77; 95% CI: 0.58–0.99). Previously, Satia *et al*.^13^ already observed in an exploratory analysis within the VITamins And Lifestyle (VITAL) study that use of glucosamine (HR: 0.72; 95% CI, 0.54–0.98) and chondroitin sulphate (HR, 0.65; 95% CI, 0.45–0.93) supplements were associated with reduced risk of CRC after 5 years of follow-up. These results were not statistically significant when adding two years of follow-up (HR: 0.55; 95 % CI 0.30–1.01)^14^. Despite the fact that we could not find an association of CG with CRC risk that was independent of NSAID use, all the above-mentioned studies^13,15^ did control for ASA and NSAIDs and they did find an independent effect. In fact, our meta-analysis of the VITAL study^14^, the Nurses’ Health Study and Health Professionals follow-up study^15^ and the MCC-Spain showed a significant overall effect, multivariate-adjusted, which was also significant both for concurrent non-NSAID users and for NSAID users (Fig. 1). The lack of heterogeneity among the studies reinforced the observed protective effect.

Though glucosamine and chondroitin sulphate have anti-inflammatory effect, its mechanism is independent of cyclooxygenase-2 inhibition and the anti-inflammatory mechanism is thought to be independent of NSAIDs. However, our stratified analysis by NSAID use does not seem to support this, as the inverse association with chondroitin sulphate and glucosamine was seen only among NSAID users. Because of the low number of CG users, we could not analyse duration and time exposure, so we could not differentiate concomitant and sequential exposure of CG and NSAIDs. We do not know if the increased protection of CG and non-ASA NSAIDs or ASA use was because of CG itself or because this subgroup used a higher dose or longer period use of NSAIDs.

*In vitro* and animal studies^14,15,21–24^ suggest that this protective effect might be caused through reduction in inflammation^25–27^ by the suppression of the NF-kβ pathway^16^, this alternative mechanism is relevant and explains the better toxicity profile of glucosamine and chondroitin sulphate observed in multiple clinical trials^28^. We should highlight that glucosamine increases insulin resistance in skeletal muscle and diabetics should take caution when taking it, however, alteration of glucose homoeostasis was not found in a 3-year randomised controlled study in patients without diabetes^29^. Moreover, some preparations that contain glucosamine extracted from seafood could increase the risk of hypersensitivity reactions among people with an allergy to shellfish^30,31^. A 2006 Cochrane systematic review^32^ concluded that glucosamine is as safe as placebo and Matheson *et al*.^33^ reported less gastrointestinal symptoms, skin reactions or fatigue with glucosamine than ibuprofen. As for chondroitin sulphate, it is considered to be safe, with rare incidence of adverse reactions which suggests its long term safety^28,31,34^. Only mild gastrointestinal side effects such as nausea, diarrhoea or constipation, stomach pain, and heart burn have been reported^34^.

Previous studies^13,14^ had controversial results of the association of chondroitin sulphate and glucosamine to CRC according to BMI. We did not find any evidence of an effect modification of CG by BMI, though we had limited power to detect this interaction. The OR for CG was 0.67 (95%CI: 0.29–1.56) for BMI ≥25kg/m^2^ and 0.76 (95%CI: 0.37–1.48) for BMI <25kg/m^2^.

This study has several limitations that might explain the low prevalence of use of chondroitin sulphate and glucosamine (1.63%). First, drug consumption was self-reported, which could introduce a recall bias and attenuate the association observed. Although we wanted to recollect detailed information of dosages and prescription duration, we could not analyse the dose-effect relationship because patients did not provide enough detailed data regarding drugs consumption. The reported prevalence of use in the USA was 13%^15^, a country in which these drugs can be self-purchased as nutritional supplements, while in Spain a prescription is required. Also, the mean age of our population (64.6 years, range 22–85) is a few years younger and with less women (44.4%) than previous studies^14,15^, which reduces the prevalence of subjects with osteoarthritis. In fact, it is reported that the highest prevalence of knee pain is amongst women aged 75^35^. There could be also surveillance bias as patients with osteoarthritis are in the same age range as CRC. Patients with osteoarthritis have regular medical visits that could result in increased screening for CRC, early intervention for polyp removal and prevention of actual CRC. Another concern is the confounding by association with NSAIDs as chondroitin sulphate and glucosamine are used essentially for osteoarthritis, and generally with NSAIDs, so the univariate analysis could essentially represent the effect of concomitant NSAIDs that have already demonstrated a protective effect^7,36^. The fact is that in the very few patients without NSAIDs there is no effect of CG on CRC. The increased effect when CG is associated with NSAIDs could simply be a dose effect of NSAIDs, if CG use selected patients with more severe osteoarthritis (a higher dose or longer period use of NSAIDs). To address these limitations, larger studies, preferably with prospective design and with exposure assessment based on registered data, should be performed.

In conclusion, we have not found clear evidence of an independent preventive effect of CG on CRC because the observed effects of our study could be attributed to NSAIDs concurrent use. However, the good toxicity profile merits further research to examine their effect and a potential role as chemopreventive agents.
